# Latest in Cellular Pathology Research

**DOI:** 10.3390/cells11244037

**Published:** 2022-12-14

**Authors:** Dimitrios Karamichos

**Affiliations:** 1 North Texas Eye Research Institute, University of North Texas Health Science Center, Fort Worth, TX 76107, USA; dimitrios.karamichos@unthsc.edu; 2 Department of Pharmaceutical Sciences, University of North Texas Health Science Center, Fort Worth, TX 76107, USA; 3 Department of Pharmacology and Neuroscience, University of North Texas Health Science Center, Fort Worth, TX 76107, USA

The year 2021 marked the 10th anniversary of the publication of Cells. To celebrate this milestone, a Special Issue entitled “10th Anniversary of Cells—Advances in Cellular Pathology” was launched. The goal of this Special Issue was the collection of impactful research/review articles in the Cellular Pathology field. The final roster of published articles for the Special Issue is an incredible collection of research articles and reviews, covering topics from cancer, diabetes, and ocular manifestations, all of which are truly consequential to the quality of life.

One of the most common types of primary liver cancer is hepatocellular carcinoma (HCC). Recurrence in HCC after conventional treatments remains a significant clinical challenge, despite advanced targeted therapies. Asadian et al. [1] reported that radionuclide therapy (^188^ReO_4_) could potentially become a new therapeutic agent against HCC. It appears that the therapy regulates the induction of apoptosis and cell cycle arrest and the inhibition of tumor formation. It is certainly an intriguing concept, and further studies are warranted in order to achieve a selective/personalized HCC therapy.

The study reported by Makboul et al. [2] focused on the histopathologic diagnosis of prostate cancer (PCa). PCa manifestation presents as a small, walnut-sized gland that produces seminal fluid in the prostate. This study explored and compared the expression of Transmembrane O-Mannosyltransferase-Targeting Cadherins 4 (TMTC4) in PCa cells and tissue specimens, evaluating its sensitivity and specificity. The authors reported high TMTC4 expression in PCa cells and tissues, with the ability, sensitivity, and specificity to differentiate between PCa and benign prostatic hyperplasia (BPH). These data are only the beginning, and if confirmed, they could be carried over to clinical practice. The lack of reliable biomarkers to diagnose PCa remains problematic due the high prevalence of the disease.

Liu et al. [3] reported their findings about ovarian cancer, another potent type of cancer. It often goes undetected until it has spread within the pelvis. At this late stage, the highly therapy-resistant cancer is a major hindrance for therapeutic efficacy, leading to higher rates of fatality. NADPH oxidase 4 (NOX4) is responsible for higher NADPH activity to increase reactive oxygen species (ROS) production. The authors found that higher levels of NOX4 were detected in a large portion of human ovarian cancer samples, suggesting a potential new mechanism in overcoming therapeutic resistance of ovarian cancer in the future.

In regard to thyroid cancer, the most prevalent endocrine malignancy, Singh et al. [4] published a review article summarizing the most recent genetic aberrations in the development and progression of thyroid cancer and implications for immunotherapy. The etiology of thyroid cancer is poorly understood and likely stems from both environmental and genetic cues. Interestingly, the disease mostly comprises indolent differentiated cancers (DTCs) and less frequently comprises aggressive poorly differentiated (PDTC) or anaplastic cancers (ATCs), with high incidence of mortality. Fortunately, in the United States, the 5-year survival rate is currently 98% for those with thyroid cancer. However, survival rates are based on various factors, including the specific type of thyroid cancer and stage of disease.

Van Acker et al. [5] reviewed the complex nature of Pterygium, an abnormal outgrowth of the conjunctiva. As stated by the authors, Pterygium is a multifaceted pathology that displays apparent conflicting characteristics: benign (e.g., self-limiting and superficial), bad (e.g., proliferative and potentially recurrent) and ugly (e.g., signs of preneoplastic transformation). This review article provides an overview of our pathophysiological understanding of benign pterygium pathology, overlapping with ocular surface squamous neoplasia and skin cancer, and highlighting considerable similarities between pterygium and other UV-related malignancies at the molecular level.

McKay et al. [6] provided an insight into the role of sex hormones and ocular pathologies, including dry eye disease and keratoconus. In this review, the authors discussed key findings that have revealed a role for androgens and estrogens in the cornea, with focus on ocular surface homeostasis, wound healing, and stromal thickness. Notably, while it is unclear if endogenous hormones contribute to differential corneal wound healing in common animal models, the abundance of evidence suggests that systemic hormone levels should be considered in studies of corneal health and disease.

Diabetes mellitus (DM) was also covered in this Special Issue. DM is one of the principal manifestations of metabolic syndrome and its prevalence with modern lifestyle is increasing perpetually. Dewanjee et al. [7] published a comprehensive review article, hinging on substantial evidence that histone deacetylase (HDAC) isoforms regulate various molecular activities in DM via epigenetic and post-translational regulation of several transcription factors. This review sheds light on the emerging role of HDACs/isoforms in DM pathophysiology and focuses on novel interventional strategies for metabolic disorders/complications that could benefit from HDACs in the future.

Extracellular vesicles (EVs) are secreted from cell membranes within the circulatory system and body fluids. Current knowledge about the involvement of EVs in numerous diseases is increasing at an ever-accelerating rate. D’Alessandro et al. [8] investigated the role of EVs in the context of idiopathic pulmonary fibrosis (IPF). IPF is a condition of scarring in the lungs, making breathing increasingly difficult as the disease progresses. It typically affects those who are 70–75 years old and rarely affects those under 50 years old. The authors aimed to evaluate the expression of exosomal surface epitopes in a cohort of IPF patients and how they may differ from their healthy counterparts. For the first time, expression of surface epitopes on EVs from IPF patients is shown, providing new insights into the pathogenesis of the disease.

Ouyang et al. [9] focused on acute pancreatitis (AP), a severe and potentially fatal disease caused predominantly by the formation of gallstones or excessive alcohol consumption. In 4 out of 5 cases, AP improves quickly and does not cause serious problems; however, 1 in 5 cases becomes severe and can result in life-threatening complications. The role of Receptor-Interacting Protein Kinase 1 (RIPK1), a key component of programmed necrosis, is unclear in AP. The authors assessed the effects of RIPK1 inhibitor Necrostatin-1 (Nec-1) and RIPK1 modification (RIPK1K45A:kinase dead) in bile acid (TLCS-AP), alcoholic (FAEE-AP) and caerulein hyperstimulation (CER-AP) mouse models. The data demonstrated the protective actions of Nec-1 in multiple AP models; however, RIPK1-dependent necroptosis only partially contributed to beneficial effects. Thus, further studies are warranted to delineate the role of Nec-1 in AP.

Finally, Iwahashi et al. [10] reported on pregnancy-specific syndrome, preeclampsia. This is a condition that plays a significant role in global maternal mortality. The classic triad of preeclampsia is hypertension, proteinuria, and edema. However, edema is no longer considered critical to this condition. Endoplasmic reticulum (ER) stress has been implicated in the pathology of preeclampsia and serves as the major risk factor. Herein, the authors examined the mechanism by which ER stress exposes the placenta to the risk of preeclampsia. Their results provided evidence that induction of ER stress leads to extracellular calreticulin (CRT) release, which may ultimately contribute to placental dysfunction.
